# Characterization of Novel Pathogenic Variants Causing Pyridox(am)ine 5′-Phosphate Oxidase-Dependent Epilepsy

**DOI:** 10.3390/ijms222112013

**Published:** 2021-11-06

**Authors:** Anna Barile, Philippa Mills, Martino L. di Salvo, Claudio Graziani, Victoria Bunik, Peter Clayton, Roberto Contestabile, Angela Tramonti

**Affiliations:** 1 Istituto di Biologia e Patologia Molecolari, Consiglio Nazionale delle Ricerche, 00185 Rome, Italy; anna.barile@uniroma1.it; 2 Dipartimento di Scienze Biochimiche “A. Rossi Fanelli”, Sapienza Università di Roma, 00185 Rome, Italy; martino.disalvo@uniroma1.it (M.L.d.S.); graziani.1761350@studenti.uniroma1.it (C.G.); 3 Genetics and Genomic Medicine, UCL Great Ormond Street Institute of Child Health, London WC1N 1EH, UK; p.mills@ucl.ac.uk (P.M.); peter.clayton@ucl.ac.uk (P.C.); 4 Belozersky Institute of Physico-Chemical Biology, Faculty of Bioengineering and Bioinformatics, Lomonosov Moscow State University, 119991 Moscow, Russia; bunik@belozersky.msu.ru; 5 Department of Biochemistry, Sechenov University, 119991 Moscow, Russia

**Keywords:** pyridox(am)ine 5′-phosphate oxidase, pyridoxal 5′-phosphate, neonatal epileptic encephalopathy, PNPO deficiency

## Abstract

Several variants of the enzyme pyridox(am)ine 5′-phosphate oxidase (PNPO), responsible for a rare form of vitamin B_6_-dependent neonatal epileptic encephalopathy known as PNPO deficiency (PNPOD), have been reported. However, only a few of them have been characterised with respect to their structural and functional properties, despite the fact that the knowledge of how variants affect the enzyme may clarify the disease mechanism and improve treatment. Here, we report the characterisation of the catalytic, allosteric and structural properties of recombinantly expressed D33V, R161C, P213S, and E50K variants, among which D33V (present in approximately 10% of affected patients) is one of the more common variants responsible for PNPOD. The D33V and E50K variants have only mildly altered catalytic properties. In particular, the E50K variant, given that it has been found on the same chromosome with other known pathogenic variants, may be considered non-pathogenic. The P213S variant has lower thermal stability and reduced capability to bind the FMN cofactor. The variant involving Arg161 (R161C) largely decreases the affinity for the pyridoxine 5′-phosphate substrate and completely abolishes the allosteric feedback inhibition exerted by the pyridoxal 5′-phosphate product.

## 1. Introduction

Pyridox(am)ine 5′-phosphate oxidase deficiency (PNPOD; OMIM: 610090) is an autosomal recessive inborn error of metabolism caused by mutations in the gene encoding pyridox(am)ine 5′-phosphate oxidase (PNPO). Patients with this disorder normally present in the neonatal period with seizures that are refractory to conventional anticonvulsant treatments but respond to treatment with supraphysiological doses of pyridoxal 5′-phospate (PLP). More recently the seizures of a subset of these patients have been shown to respond to pyridoxine (reviewed by [1]). PNPO is a fundamental FMN-dependent enzyme that produces PLP by oxidizing pyridoxine 5′-phosphate (PNP) and pyridoxamine 5′-phosphate (PMP) recycled from non-pyridoxal B_6_ vitamers in the diet and generated by metabolism and protein turnover [2]. PLP, the catalytically active form of vitamin B_6_, is the cofactor for about 4% of all known cellular enzymes [3], many of which are involved in the biosynthesis and metabolism of neurotransmitters and neuromodulators in the brain, including dopamine, γ-aminobutyric acid (GABA), serotonin, histamine, D-serine, and epinephrine [1]. Analysis of metabolites present in cerebrospinal fluid and in urine of patients with PNPOD has shown reduced activity of PLP-dependent enzymes involved in neurotransmitter metabolism [1].

PNPO also plays an important regulatory role in PLP homeostasis. A recent detailed biochemical characterisation of recombinant human PNPO has revealed an inhibition mechanism brought about by PLP, the reaction product of the enzyme, which binds to an allosteric site. Binding of substrate at the active site and binding of PLP at the allosteric site influence each other, increasing the respective dissociation constants. When the enzyme binds both the substrate at the active site and PLP at the allosteric site at the same time, it retains the capability to catalyse the conversion of the substrate into product, albeit at a lower rate [4].

To date, 27 pathogenic mutations of the gene encoding human PNPO have been genetically confirmed [1], of which 15 are missense variants affecting 11 different PNPO amino acid residues. Some of these missense variants, i.e., R229W [5], R95C [6], R116Q [7], G118R, R141C, R225H, R116Q/R225H, and the termination codon variant X262Q [4], have been recombinantly expressed and biochemically characterized, giving insight into the important role these variants play in PNPO activity. However, D33V, one of the more common variants responsible for PNPOD (present in approximately 10% of patients), has yet to be studied in detail. This paper describes the biochemical characterisation of the catalytic, allosteric and structural properties of recombinantly expressed D33V. The R161C, P213S, and E50K variants, which have not been previously characterized biochemically, were an object of study. The mutation causing the E50K variant, which has only been found on the same chromosome (i.e., in *cis*) with other known pathogenic variants, and has been reported as non-pathogenic, has recently been commented on by Alghamdi et al. (2020) [8] to cause severe disease. The biochemical characterization of the recombinant variants, together with the clinical study of patients is important in order to optimize treatment of the disease, i.e., to predict whether the patient will respond to pyridoxine or will need PLP or whether riboflavin supplementation to increase FMN levels will enhance PNPO activity.

## 2. Results

### 2.1. Physical Properties of PNPO Variants

**Figure 1:**
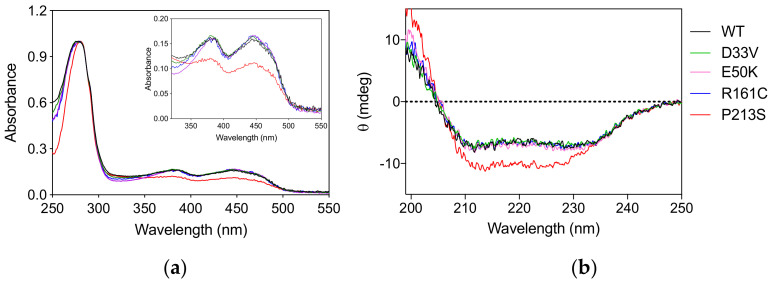
Spectroscopic properties of PNPO variants. (**a**) Absorption spectra of PNPO variants. The inset is an expanded view of the visible region of the same absorption spectra, showing the absorption bands due to FMN. (**b**) Far-UV CD spectra of PNPO variants. The absorption and CD spectra of the wild type enzyme are also reported as reference. All spectra were measured in 20 mM potassium-phosphate buffer, pH 7.6.

The PNPO variants D33V (rs370243877), E50K (rs549477447), R161C (rs146027425), and P213S were produced and purified following the protocol used for the wild type enzyme (Materials and Methods). Size-exclusion chromatography showed that all the variants have the same elution profiles as the wild type enzyme (Figure S1), corresponding to the dimeric form of the protein. The absorption spectrum and the amount of FMN bound to the variant forms, quantified on the basis of the absorbance at 445 nm [9] measured at the end of the purification procedure, showed that all the variants, except P213S, were fully saturated with the cofactor (Figure 1a). The absorption spectrum of the P213S variant suggests that it has a reduced ability to bind FMN, with only 65% of enzyme bound to FMN. Circular dichroism (CD) spectra of D33V, E50K, and R161C variants showed no significant secondary structure differences compared to wild type, whereas that of the P213S variant shows an increased negative signal (Figure 1b).

**Figure 2:**
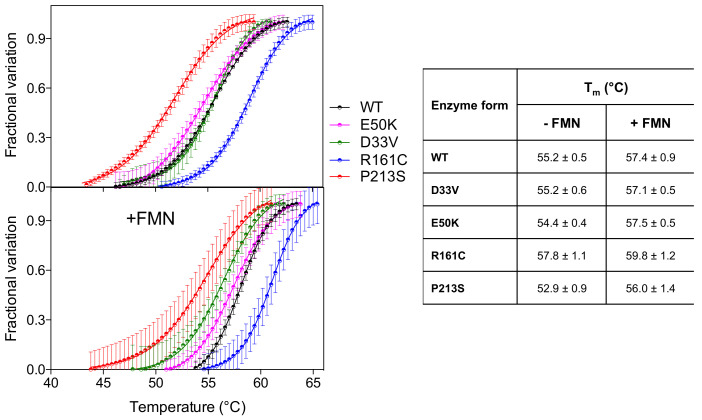
Temperature dependence of fluorescence change is expressed as fractional variation for the wild type PNPO and all variants (2 µM) indicated in the legend. The purified variants are tested without and with an equimolar amount of FMN (+FMN). The curves shown in the figure are the average with standard error bars of three independent experiments. Data are fitted to the Boltzmann equation to obtain the melting temperatures (listed in the table on the right).

Analysis of the thermal stability of the D33V and E50K variants, using differential scanning fluorimetry (DSF), revealed that these were similar to the wild type enzyme (Figure 2) (T_m_ ~ 55 °C). The R161C variant, however, was more stable (T_m_ = 57.8 ± 1.1 °C) relative to the wild type, whereas the P213S variant was less stable (T_m_ = 52.9 ± 0.9 °C). The presence of an equimolar amount of FMN stabilizes all the PNPO forms by increasing the T_m_ by 2–3 °C.

### 2.2. Kinetic and Allosteric Properties of PNPO Variants

Kinetic measurements (K_M_ and k_CAT_) for the PNPO variants were carried out using PNP as substrate by following the formation of the aldimine complex between the PLP reaction product and Tris (Table 1 and Figure S2). The assays were performed in 50 mM Tris–HCl buffer pH 7.6 at 37 °C in the presence of a molar excess of FMN (five times the enzyme concentration), to ensure that the enzyme was completely saturated with the cofactor. The D33V, E50K, and P213S PNPO variants show increased values of K_M_ for PNP, whereas the k_CAT_ values remain almost unchanged with respect to wild type. The R161C variant shows a very large K_M_ (340-fold larger than the wild type), whereas k_CAT_ is only 3.5-fold lower than that of the wild type. This variant, just as the wild type enzyme, proved to be completely inactive with PN as substrate.

**Figure 3:**
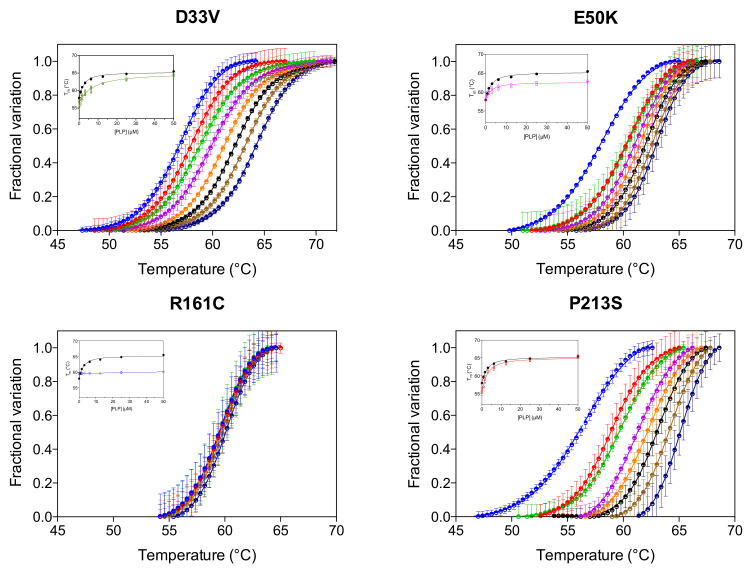
PLP binding at the active site. DSF measurements with PNPO variants in the presence of different PLP concentrations. Fluorescence change is expressed as fractional variation as a function of temperature. The experiment was carried out using 2 µM enzyme and different PLP concentrations (0, 0.78, 1.56, 3.13, 6.25, 12.5, 25 and 50 µM), which are displayed in different colours from light blue (0 µM) to dark blue (50 µM). Thermal denaturation data are fitted to the Boltzmann equation to obtain melting temperatures. Each curve is the average of three independent experiments with standard error bars. In the insets, the saturation curves obtained by plotting the melting temperatures as a function of the PLP concentration are shown. The saturation curve of the wild type enzyme (in black) is reported as reference. Data were analysed using Equation (1) to estimate dissociation constant values (K_D2_) (reported in Table 1).

We have previously reported that the thermal stability of wild type PNPO increases upon binding PLP at the active site and that the analysis of the melting temperature as a function of PLP concentration gave a dissociation constant value (K_D2_, Table 1) that roughly corresponds to the K_M_ value for the PNP substrate [4]. K_D2_ was also measured for the variants (Figure 3, Table 1). As for wild type and PNPO variants characterized previously [4], the K_D2_ value of the D33V variant is very similar to the K_M_ for PNP. Conversely, the K_D2_ values of E50K and P213S variants are smaller than their respective K_M_, suggesting a better binding of the product PLP than the substrate PNP. These K_D2_ values are very similar to those of the wild type enzyme (Table 1). PLP does not seem, however, to bind at the active site of the R161C variant.

**Figure 4:**
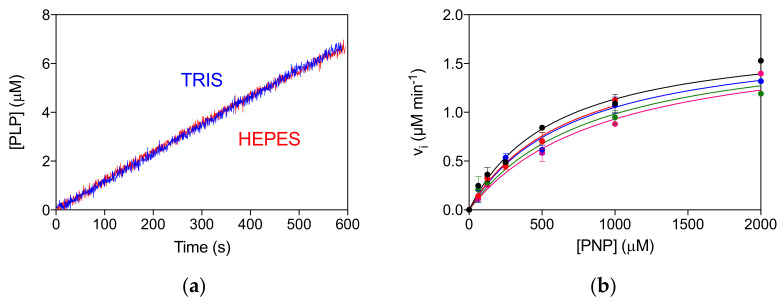
Lack of allosteric properties of the R161C variant. (**a**) Comparison of kinetics carried out in 50 mM Tris–HCl and 50 mM Na–HEPES buffers at pH 7.6, obtained using 2 μM enzyme and 500 μM PNP. (**b**) The initial velocity of the reaction was measured with 2 µM enzyme (protein subunit concentration), varying PNP concentration while keeping exogenous PLP fixed at different concentrations (0, 0.5, 1, 2, and 4 µM). Data are the average ± standard deviation of three independent measurements. The resulting saturation curves were fitted to the Michaelis–Menten equation, obtaining very similar apparent V_max_ and K_M_ values at all PLP concentrations.

The allosteric properties of the PNPO variants were also investigated by fluorimetric analysis of PLP equilibrium binding (Figure S3A, Table 1). The high affinity K_D_ values (K_D1_) for the D33V, E50K and P213S pathogenic variants were shown to be similar to that of the wild type enzyme, suggesting that PLP binding at the allosteric site is unaffected. The unchanged allosteric properties are also evident from the comparison of reaction kinetics measured in Tris and Hepes buffer (Figure S3B), which is similar to that found for the wild type enzyme [4]. In particular, while the kinetics of PLP production in Tris is linear, since the PLP produced is sequestered by Tris, in Hepes buffer PLP accumulates in the solvent, binds to PNPO at the allosteric site and inhibits the enzyme [4,10]. The R161C variant shows a smaller K_D1_ value with respect to the wild type PNPO (Figure S3A, Table 1). Moreover, the reaction kinetics in Tris buffer is superimposable to that in Hepes buffer, suggesting a lack of PLP feedback inhibition (Figure 4a). For this variant, a complete inhibition kinetic characterisation was carried out in Hepes buffer. The initial velocity of the reaction was measured by varying PNP concentration at different, fixed exogenous PLP concentrations, using 2 μM enzyme (Figure 4b). The analysis of saturation curves confirmed the lack of inhibition for this variant.

## 3. Discussion

Table 2 summarises the PNPOD cases reported in the literature concerning the variants analysed in this study. Classification of the neurodevelopmental outcome as “Mild”, “Normal” or “Severe” was based on the clinical course of the disease described in the original publications and reported in Table S1. The neurodevelopmental impact of the disease is likely determined by early diagnosis and treatment rather than genotype. For the majority, whilst vitamin B_6_ treatment needs to be life-long, the neurodevelopmental outcome was good (provided treatment was started promptly after the onset of seizures). However, the biochemical characterization of the recombinantly expressed variants yielded new insights into the molecular basis of the disease.

Three patients were homozygous for the D33V variant (1–3 in Table 2) and four others were compound heterozygotes for D33V and another variant (4–7 in Table 2); 5/7 had mild neurodevelopmental disorders and 1/7 a normal outcome (Table 2). Only one patient (5 in Table 2) who was compound heterozygote for D33V and c.264-21_264-1delinsC had severe developmental delay [12] whether this is due to the deletion/insertion event or the length of time taken to control their seizures is not possible to determine. Previous in vitro expression studies of the D33V variant using a HeLa cell lysate-based system showed reduced PNPO activity (44% relative to wild type) when using PMP as a substrate as in Mills et al. (2014) [12]. Our biochemical characterization showed only an increase in K_M_ for PNP with respect to the wild type enzyme (Table 1), which may explain a decrease in PNPO activity under some conditions, such as low substrate concentration. The D33 residue is not visible in the electron density map of the human PNPO crystal structure (PDB: 1NRG). It is therefore hard to predict what sort of effect this variant may have on the structural and functional properties of the enzyme.

**Figure 5:**
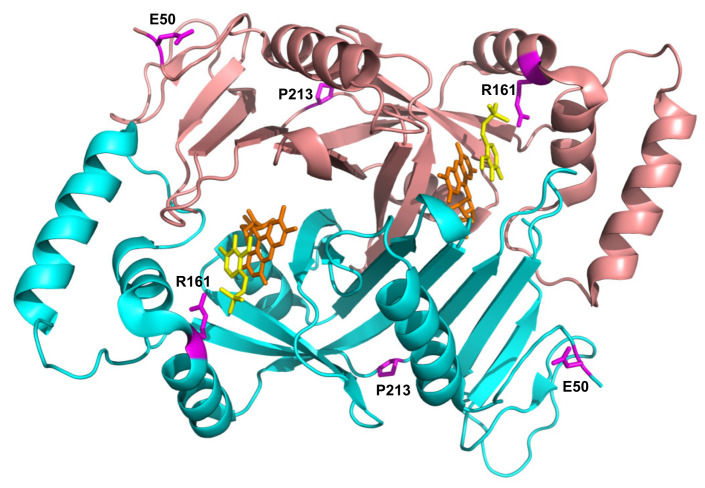
Location of the residues involved in novel pathogenic variants in PNPO structure. Crystallographic structure obtained in the presence of PLP (PDB: 1NRG; [17]). FMN and PLP in the active site are shown as sticks, in orange and yellow colour, respectively. The E50, R161, and P213 residues are shown as magenta sticks. D33 is not present in the electron density map, hence is not visible in the structure.

E50K has never been found in homozygous form (Table 2), and previous reports conflict as to the pathogenicity of this variant. A recent review by Alghamdi et al. (2020) [8], predicting the effects of PNPO variants on function, suggested that E50K causes only a mild loss of stabilizing surface interactions, but classified it as a severe mutation based on the clinical outcome of patients 8 and 9 (Table 2) with this variant. Previous studies, however, had shown that it was likely not to be pathogenic [14]. The severe outcomes observed in patients 8 and 9 (Table 2) are highly likely to be due to the splice error found associated in *cis* with the E50K variant (as is also the case for patient 10), and the late diagnosis, respectively. Additionally, of note, patient 9 (compound heterozygote for E50K and R116Q) was analysed by targeted next generation sequencing using a custom-designed panel; it is possible that a large deletion or a deep intronic variant may not be detected using this approach. Our biochemical characterization of the recombinant E50K variant showed only an increase in K_M_, whereas k_CAT_ and other properties (thermal stability, allosteric binding of PLP) remained unchanged (Table 1 and Figure 2). In vitro expression experiments in CHO cells have also shown that E50 K PNPO activity was similar to that of wild type [14] and when expressed using an IVT and HeLa cell lysate system [12] there was only a decrease in 25% in activity relative to wild type. The E50 residue is the second visible residue at the N-terminal end of the electron density map of the human PNPO crystal structure. It is located on the solvent-exposed protein surface, far from the active site, and it does not seem to play any functional or structural role (Figure 5).

Characterization of the recombinant R161C variant revealed a large increase in K_M_ for PNP, which corresponds to a drastic decrease in affinity for the substrate, but with a relatively small decrease in k_CAT_. This is not surprising, given the position of the R161 residue (Figure 5) within the active site of the enzyme, interacting with the phosphate moiety of the substrate [5]. The striking observation with this variant is the abolition of the allosteric feedback inhibition exerted by PLP, which in the wild type enzyme is responsible for a drastic decrease in activity even at PLP concentrations in the low micromolar range [4]. Nevertheless, PLP binds even tighter at the allosteric site of the R161C variant with a K_D_ that is half that of the wild type enzyme (Table 1 and Figure S3). This may be due to the R161C variant abolishing the negative allosteric coupling between the active site and the allosteric site [4]. The mild outcome observed in patient 12, who is homozygous for this variant [15] and has responded well to pyridoxine supplementation (Table 2), may be explained by the fact that, although the R161C variant K_M_ is much larger than the wild type, the k_CAT_ is only marginally decreased. Hence, provided that the PNP cellular concentration (deriving from the phosphorylation of PN acted by PDXK) is sufficiently high, the R161C variant can produce PLP at an adequate rate. Supporting this hypothesis is the observation that patient 12 responded to PN supplementation within days, after a prolonged administration of the B_6_ vitamer [15]. On the other hand, the lack of PLP feedback inhibition may be deleterious if high PN doses are administered and PLP accumulates, reaching toxic concentrations. Another component of the observed response of patient 12 to PN (and of any other patient who is PN-responsive) may involve participation of the gut flora. In a model system in which *C. elegans* is given a gut flora that comprises a single strain of *E. coli* that is PNPO deficient (Δ*pdxH*), the host worm develops features of PLP deficiency [18]. This suggests that the gut flora contributes to holobiont PLP homeostasis.

The P213S variant was found in two siblings [16]. The first, who developed epilepsy, responded to treatment with PLP after no response to pyridoxine. The mother took a pregnancy multivitamin preparation containing pyridoxine whilst pregnant with her second child and was given an additional PLP supplement just prior to their sibling’s diagnosis. The baby then received PLP from birth. Although patient 15 has had two seizures, their neurological development is normal (Table 2). The P213 residue is located on a loop connecting the two sections of the S6 β-strand (Figure 5) described by Musayev et al. [17] in the crystal structure of human PNPO. The replacement of this proline residue with a serine affects the structure and stability of the protein with an impact on the substrate affinity (Figure 1, Figure 2 and Table 1). The recombinant P213S variant was less stable than the wild type enzyme (Figure 2), bound less FMN (Figure 1) and the K_M_ was increased with respect to the wild type PNPO. Given the low affinity for FMN of this variant, PNPOD patients with this mutation may benefit from riboflavin supplementation to improve enzyme activity. The normal neurodevelopmental outcome reported for the patients carrying this variant is likely due to prompt administration of PLP at day 3 and from birth to patients 14 and 15, respectively [16].

The biochemical characterisation of variants is important, giving insight into the disease mechanism and therefore the potential of improving treatment. Treatment of PNPOD with high dose vitamin B_6_ controls seizures far better than antiepileptic drugs and can prevent the devastating outcomes of uncontrolled epileptic encephalopathy, death and severe neurodevelopmental disability. However, treatment is far from perfect. Some patients require very high doses of PLP administered frequently to prevent symptoms. Some patients on high dose PLP treatment have deranged liver function tests progressing to cirrhosis [19] and liver failure or hepatocellular carcinoma (unpublished observation). In some patients, high dose pyridoxine treatment controls seizures better than PLP, but other PNPOD patients have developed a severe peripheral neuropathy on high dose pyridoxine [12]. At least one patient has found that taking a vitamin supplement containing riboflavin led to an improvement in symptoms [12]; increasing the level of FMN could enhance PNPO activity in other patients. Combined treatment with riboflavin and PLP or PN may represent a good therapeutic strategy for some patients. Furthermore, PLP could be administered in the form of the PL precursor in order to lighten the metabolic load of the liver.

## 4. Materials and Methods

### 4.1. Site-Directed Mutagenesis, Expression and Purification of PNPO Variants

Site-directed mutagenesis was performed using QuickChange methodology (Stratagene, La Jolla, CA, USA). Mutagenic primers, synthesized by Metabion International AG (Steinkirchen, Germany), have the following sequences:

D33Vfor: 5′-CAGTGCTGCCATGGTGCTGGGACCCATG-3′

D33Vrev: 5′-CATGGGTCCCAGCACCATGGCAGCACTG-3′

E50Kfor: 5′-GAGGCATTTGAGAAAACTCATCTGAC-3′

E50Krev: 5′-GTCAGATGAGTTTTCTCAAATGCCTC-3′

R161Cfor: 5′-CTACTTCCACTCCTGCCCCAAGAGCAG-3′

R161Crev: 5′-CTGCTCTTGGGGCAGGAGTGGAAGTAG-3′

P213Sfor: 5′-CTATGTCCTGTACTCTCAGGTGATGGAG-3′

P213Srev: 5′-CTCCATCACCTGAGAGTACAGGACATAG-3′

Competent *E. coli* Rosetta (DE3) cells were transformed with pET28-*PNPO* constructs carrying the D33V, E50K, R161C, and P213S PNPO variants. Purification of the variant forms was carried out as described in [4].

### 4.2. Spectroscopic Measurements

All spectroscopic measurements were carried out at 20 °C in 20 mM potassium phosphate, pH 7.6. UV-visible spectra were recorded with a Hewlett-Packard 8453 diode-array spectrophotometer (Agilent Technologies, Cernusco sul Naviglio (MI), Italy). Far-UV (190–250 nm) CD spectra were measured with a Jasco 710 spectropolarimeter equipped with a DP 520 processor using 0.1 cm path length quartz cuvettes and results were expressed as ellipticity [Θ].

### 4.3. Size Exclusion Chromatography

Gel filtration of PNPO enzymes was performed on a Superdex 200 10/300 GL column (GE Healthcare, Milano, Italy) as described previously [20].

### 4.4. Differential Scanning Fluorimetry (DSF) Assays

DSF assays were performed on a Real Time PCR Instrument (CFX Connect Real Time PCR system, Bio-Rad) using 2 μM wild type and PNPO variants in 50 mM Na HEPES, pH 7.6, 150 mM NaCl, and Sypro Orange (5x, Thermo Scientific) in the presence of different concentrations of PLP (total volume of 25 μL) in a 96-well PCR plate. Fluorescence was measured from 25 °C to 95 °C in 0.4 °C/30 s steps (excitation 450–490 nm; detection 560–580 nm). All samples were run in triplicate. Denaturation profiles were analysed as described in [21] and the melting temperatures calculated. The melting temperatures were analysed as a function of PLP concentration according to the following equation.

(1) $$T_{m}=\left(T_{m}^{inf}-T_{m}^{0}\right)\frac{\left[PLP_{0}\right]+\left[E_{0}\right]+K_{D}-\sqrt{{\left(\left[PLP_{0}\right]+\left[E_{0}\right]+K_{D}\right)}^{2}-4\left[PLP_{0}\right]\left[E_{0\;}\right]}}{2\;\left[E_{0}\right]}+T_{m}^{0}$$

In this equation, *T_m_* corresponds to the observed melting temperature, $T_{m}^{inf}$ is *T_m_* at saturated PLP concentration, *T_m_*^0^ is *T_m_* in the absence of PLP, [*PLP*_0_] is the total PLP concentration and [*E*_0_] is the total enzyme subunit concentration.

### 4.5. PNPO Activity Assays

Activity assays were carried out in 50 mM Tris–HCl, pH 7.6, containing 5 mM 2-mercaptoethanol, at 37 °C. The reaction was started by the addition of PNP and kept under constant stirring by a magnetic bar to ensure a rapid mixing. The progress of the reaction was followed at 414 nm where the characteristic aldimine product PLP–Tris absorbs maximally with a molar absorbance coefficient of 4253 M^−1^ cm^−1^. Kinetic constant measurements were performed using 2 µM PNPO, and PNP concentrations between 0.5 and 480 µM. Initial velocities were determined over the first 20 s of the reaction. The values of K_M_ and *k_CAT_* were determined from least-squares fitting of initial velocity data as a function of PNP concentration to a quadratic equation (Equation (2)), in which *v_i_* is the initial velocity of the reaction, *k_CAT_*·[*E*_0_] corresponds to V_MAX_ (the maximum velocity of the reaction), [*PNP*_0_] is the total substrate concentration, [*E*_0_] is the total enzyme concentration and *K_D_* is the dissociation constant of the substrate binding equilibrium E+PNP ⇋E·PNP that, assuming a rapid establishment of the equilibrium, is equivalent to K_M_.

(2) $$v_{i}=k_{CAT}\left[E_{0}\right]\frac{\left[PNP_{0}\right]+\left[E_{0}\right]+K_{D}-\sqrt{{\left(\left[PNP_{0}\right]+\left[E_{0}\right]+K_{D}\right)}^{2}-4\left[PNP_{0}\right]\left[E_{0\;}\right]}}{2\;\left[E_{0}\right]}$$

### 4.6. Analysis of PLP Binding Equilibrium

Analyses of PLP binding, based on the FMN fluorescence increase observed upon binding of PLP to PNPO, was performed as described in [4].

## Figures and Tables

**Table 1:** Parameters obtained from kinetic and equilibrium measurements of PNPO variants using PNP as a substrate and PLP as an allosteric effector.

	Kinetic Parameters		PLP Binding	
Enzyme Form	K_M_ (µM)	k_CAT_ (min^−1^)	K_D1_ (µM) ^1^	K_D2_ (µM) ^1^
WT ^2^	2.6 ± 0.2	3.72 ± 0.10	0.95 ± 0.01	3.2 ± 0.2
D33V	10.3 ± 0.7	5.30 ± 0.32	1.00 ± 0.09	6.6 ± 1.3
E50K	9.5 ± 0.6	3.70 ± 0.05	0.93 ± 0.04	2.2 ± 1.0
R161C	894 ± 125	0.99 ± 0.07	0.53 ± 0.08	ND
P213S	32.0 ± 2.0	4.72 ± 0.08	1.94 ± 0.10	2.4 ± 0.8

^1^ K_D1_ is the dissociation constant for binding of PLP to the high affinity (allosteric) site and K_D2_ is the dissociation constant for binding to the lower affinity (active) site. ^2^ from [4]. ND, not determined.

**Table 2:** Summary of medical observations of patients with the variants characterized in this work.

N	Variant (Inheritance)	Seizure on Set ^1^	Seizure Response to PN ^2^	Seizure Response to PLP ^2^	Neurodevelopmental Outcome ^3^	Reference
1	D33V (Homozygote)	4 weeks	−	+	Mild	P4 [11]
2	D33V (Homozygote)	6 h	−	+	Mild	P2 [12] P3 [3]
3	D33V (Homozygote)	3 weeks	+	Frequency increased compared to PN	Mild	P9 [12]
4	D33V; R116Q + R225C ^3^ (Compound Heterozygote)	2 weeks	+	Not trialled	Mild	P12 [12]
5	D33V + c.264-21_264-1 delins C (Compound Heterozygote)	3h	+	+	Severe	P11 [2]
6	D33V + Leu83Trpfs ^4^ 17 (Compound Heterozygote)	36 h	−	+	Normal	P3 [4] P4 [3]
7	D33V + E120K (Compound Heterozygote)	2 months	+	Not trialled	Mild	P10 [12]
8	R95H; E50K + c.364−1G > A ^5^ (Compound Heterozygote)	30 min	−	+	Severe	P1 [12]
9	E50K + R116Q (Compound Heterozygote)	40 days	+	Not trialled	Severe	P4 [13]
10	E50K + c.364−1G > A (Homozygous for both) ^6^	2 h	Not trialled	Not trialled	Severe	J1 [14]
11	E50K + c.364-1G > A (Homozygous for both) ^6^	1 h	−	+	Severe	J2 [14] sibling of 10
12	R161C (Homozygote)	2 days	+	Not trialled	Normal	[15]
13	R161C+ p. Pro150ArgfsTer27 (Compoud Heterozygote)	24 h	Reduction in seizure frequency	Reduction in seizure frequency	Severe	Twin P1 and P2 [5]
14	P213S (Homozygote)	90 min	−	+	Normal	P3 [12,16]
15	P213S (Homozygote)	No seizures, treated during pregnancy and from birth	Not trialled	+	Normal	P4 [12,16] sibling of 14.

^1^ Time post birth and without any drug. ^2^ Reduction in frequency to <10% pretreatment. ^3^ See Table S1 for further details. ^4^ D33V inherited from the paternal allele and R116Q in combination with R225C from the maternal allele. ^5^ This mutation causes splice errors. E50K and the splicing variant were in *cis*. ^6^ The patient was homozygous for both variants and the parents heterozygous, i.e., E50K and the splicing error were in *cis*.

## Supplementary Materials

The following are available online at https://www.mdpi.com/article/10.3390/ijms222112013/s1, Figure S1: Size exclusion chromatography analysis of PNPO variants, Figure S2: Catalytic properties of PNPO variants, Figure S3: Allosteric properties of PNPO variants, Table S1: Clinical course of patients with the variants characterized in this work.
