# Supplementary material for: Characterization of Novel Pathogenic Variants Causing Pyridox(am)ine 5′-Phosphate Oxidase-Dependent Epilepsy
Source: Int J Mol Sci. 2021 Nov 6;22(21):12013. doi: 10.3390/ijms222112013 (PMC8584306; doi:10.3390/ijms222112013)
Supplement: Supplementary file 1 [file ijms-22-12013-s001.zip › ijms-1452964-supplementary.pdf]

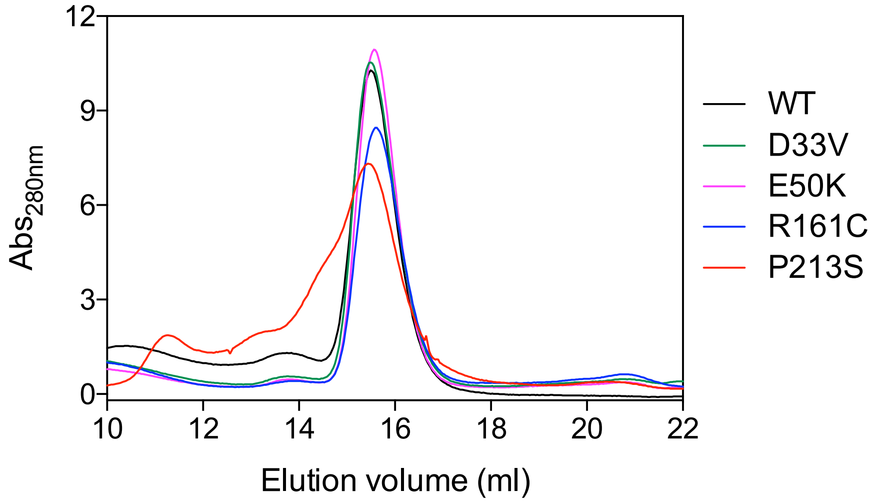

Figure S1. Size exclusion chromatography analysis of PNPO variants. Elution profiles were obtained in 20 mM potassium-phosphate buffer, pH 7.6, containing 150 mM NaCl and 5 mM 2-mercaptoethanol. All variants are in dimeric form, as shown by the elution volume of the main elution peak, although a small elution band of high molecular weight is also visible. The P213S variant also shows a small shoulder on the main elution peak, probably due to the instability of the protein.

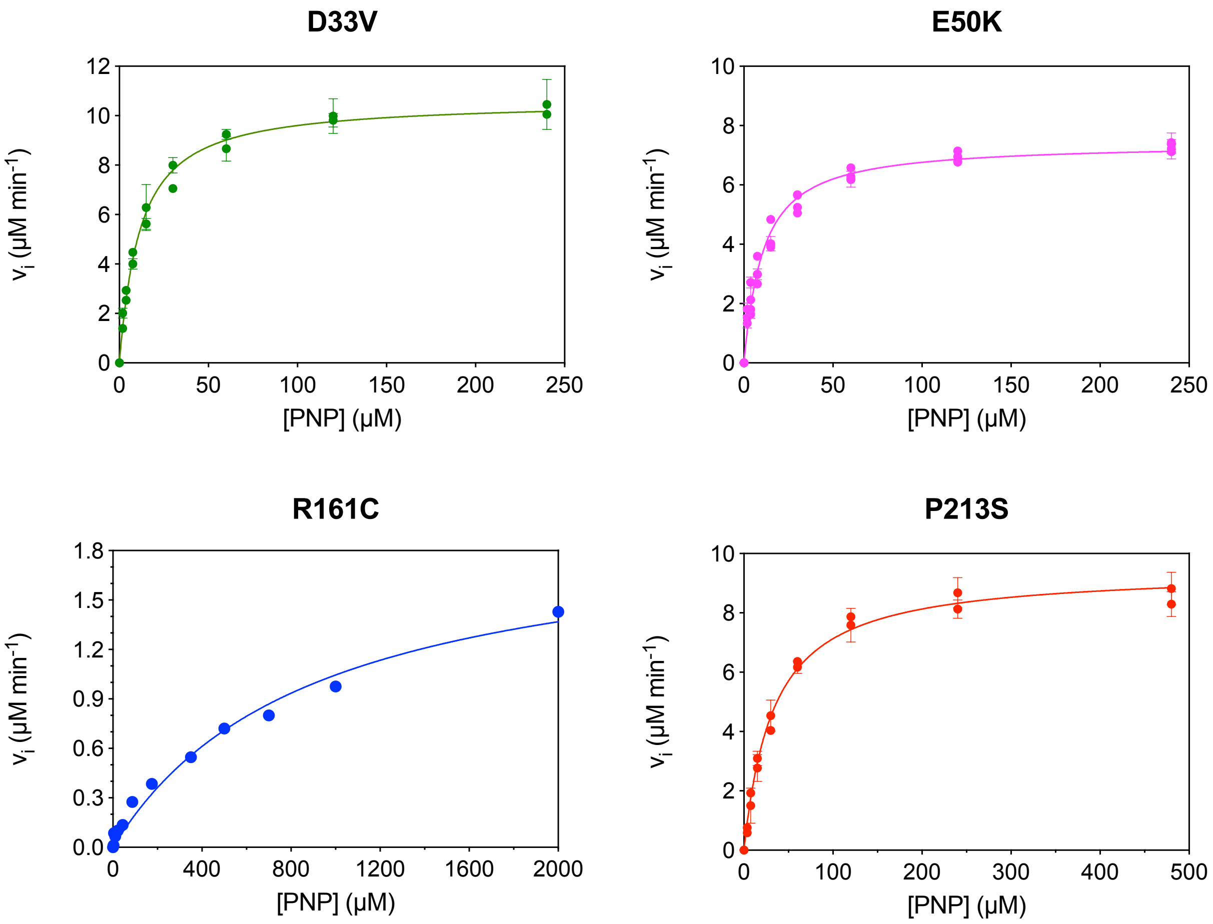

Figure S2. Catalytic properties of PNPO variants. Saturation curves representing the initial velocity as a function of PNP concentration. Continuous lines are from fitting to equation 2.

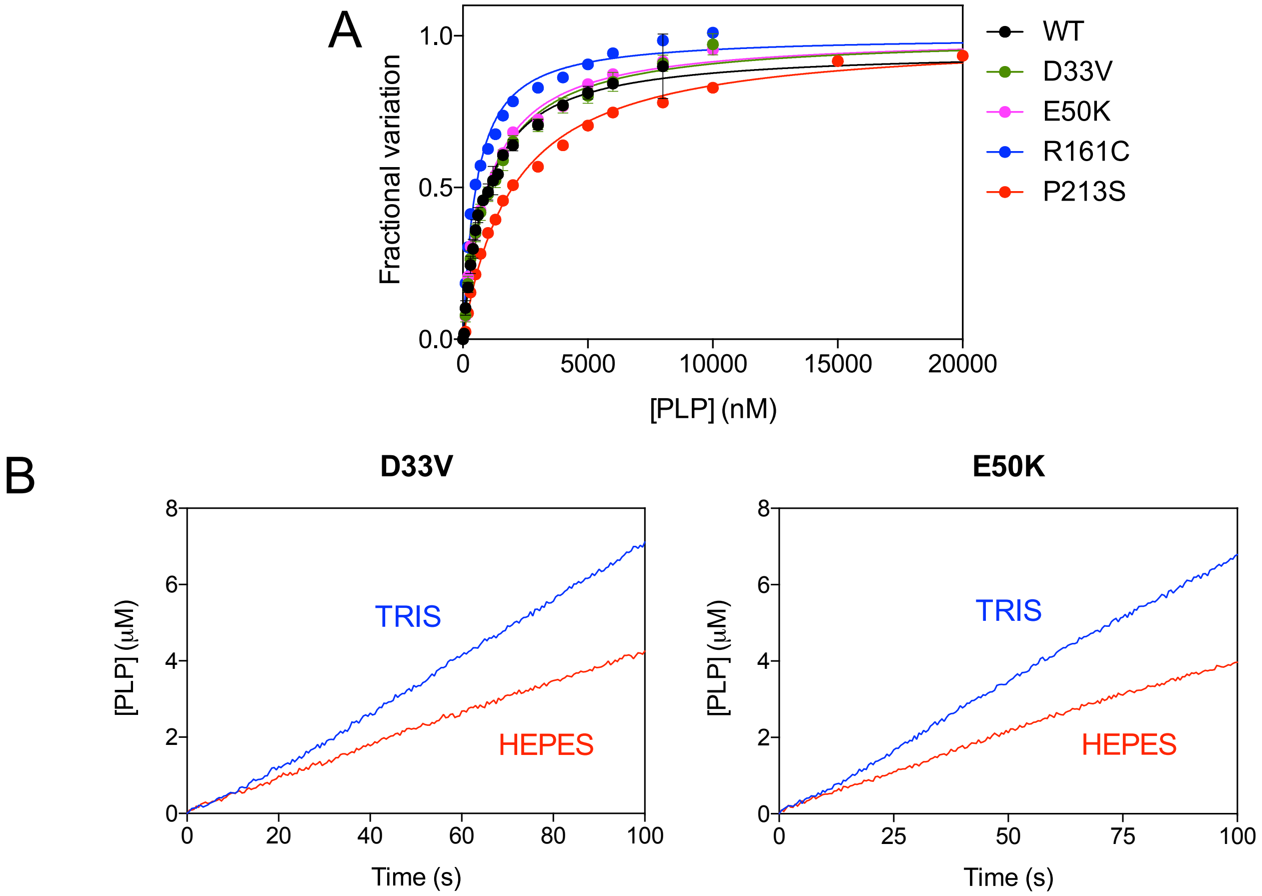

Figure S3. Allosteric properties of PNPO variants. A) Saturation curves resulting from PLP binding to the indicated PNPO forms. Fluorescence change expressed as fractional variation is plotted as a function of PLP concentration. Continuous lines are from fitting as described in Barile *et al.* 2020. B) Comparison of kinetics carried out in 50 mM Tris-HCl and 50 mM Na-HEPES buffers at pH 7.6, obtained using 1 μM enzyme and 120 μM PNP.

Table S1. Clinical course and outcome of patients with variants analysed in this work

N	**Variant** (inheritance)	**Clinical course and outcome**
1	**D33V** (Homozygote)	Good control of paroxysmal events with PLP. At 11 months she is standing, waves bye bye and is saying her first word. Only occasional seizures.
2	**D33V** (Homozygote)	Good seizure control with PLP. Development normal at age 5.
3	**D33V** (Homozygote)	Good seizure control with PLP. Neurodevelopmental outcome essentially normal: minor delay in expressive language but advanced reading ability at 7.5y.
4	**D33V; R116Q + R225C** (Compound Heterozygote)	Good seizure control with PN. Morning auras improved by a multivitamin B supplement. Intellectually normal, but with some dyslexia and features of Asperger syndrome.
5	**D33V+ c.264-21_ 264-1delinsC** (Compound Heterozygote)	Poor seizure control in the first 3 months of life and severe epileptic encephalopathy at 5y when PN was stopped.
6	**D33V+Leu83Trpfs*17** (Compound Heterozygote)	Needed high doses of PLP at frequent intervals for full seizure control. He has required a liver transplant (unpublished).
7	**D33V+ E120K** (Compound Heterozygote)	Good seizure control on PN but developed neuropathy. Mild intellectual disability requiring extra support in mainstream school.
8	**R95H; E50K + 364-1G>A** (Compound Heterozygote)	Frequent PLP required for full seizure control. Development delayed; at age 2y was crawling but not walking, had a few words but no sentences.
9	**E50K+R116Q** (Compound Heterozygote)	Seizures controlled with valproic acid and topiramate. PN added when PNPOD diagnosed. At 8y he cannot speak or look after himself, likely to be due to delay in treatment with B_6.
10	**E50K + c.364-1G>A** (Homozygous for both)	Uncontrolled neonatal epileptic encephalopathy (not treated with B_6), abdominal distension, vomiting, died at 5w.
11	**E50K + c.364-1G>A** (Homozygous for both)	Severe neonatal epileptic encephalopathy, treated with PLP from 12 days, persistent central hypotonia and by 2y severe painful dystonic spasms and seizures. He had marked acquired microcephaly and moderate to severe developmental delay.

**12**	**R161C** (Homozygote)	Good seizure control with PN. Neurodevelopmental outcome at 14m is normal.
**13**	**R161C+ p.Pro150ArgfsTer27** (Compound Heterozygote)	Took several years before PLP was trialed – seizures are not controlled.
**14**	**P213S** (Homozygote)	Good seizure control on PLP, given from 40h, although seizures can occur at trough times i.e. early in the morning or delayed administration. At 4.5y formal clinical neurological and developmental examination were normal.
**15**	**P213S** (Homozygote)	Mother given multivitamin containing PN during pregnancy and PLP during last three days of the pregnancy. PLP was then administered from birth. At 2.5y neurological examination was normal and neurodevelopment within age-appropriate parameters.
